# BCG and Alternative Therapies to BCG Therapy for Non-Muscle-Invasive Bladder Cancer

**DOI:** 10.3390/curroncol31020079

**Published:** 2024-02-16

**Authors:** Sarah Lidagoster, Reuben Ben-David, Benjamin De Leon, John P. Sfakianos

**Affiliations:** 1 Department of Urology, Ichan School of Medicine at the Mount Sinai Hospital, New York, NY 10029, USAreuben.bendavid@mountsinai.org (R.B.-D.); benjamin.deleon@downstate.edu (B.D.L.); 2 CUNY School of Medicine, City College of New York, New York, NY 10031, USA; 3 SUNY Downstate Health Science University, New York, NY 11203, USA

**Keywords:** bladder cancer, intravesical therapy, BCG failure, BCG

## Abstract

Bladder cancer is a heterogeneous disease. Treatment decisions are mostly decided based on disease stage (non-muscle invasive or muscle invasive). Patients with muscle-invasive disease will be offered a radical treatment combined with systemic therapy, while in those with non-muscle-invasive disease, an attempt to resect the tumor endoscopically will usually be followed by different intravesical instillations. The goal of intravesical therapy is to decrease the recurrence and/or progression of the tumor. In the current landscape of bladder cancer treatment, BCG is given intravesically to induce an inflammatory response and recruit immune cells to attack the malignant cells and induce immune memory. While the response to BCG treatment has changed the course of bladder cancer management and spared many “bladders”, some patients may develop BCG-unresponsive disease, leaving radical surgery as the best choice of curative treatment. As a result, a lot of effort has been put into identifying novel therapies like systemic pembrolizumab and Nadofaragene-Firadenovac to continue sparing bladders if BCG is ineffective. Moreover, recent logistic issues with BCG production caused a worldwide BCG shortage, re-sparking interest in alternative BCG treatments including mitomycin C, sequential gemcitabine with docetaxel, and others. This review encompasses both the historic and current role of BCG in the treatment of non-muscle-invasive bladder cancer, revisiting BCG alternative therapies and reviewing the novel therapeutics that were approved for the BCG-unresponsive stage or are under active investigation.

## 1. Introduction

Bladder cancer ranks as the fourth most prevalent cancer in men. Recently, the Surveillance, Epidemiology, and End Results (SEER) database collected population-based cancer incidence data in the United States and projects that 82,290 people will be diagnosed with bladder cancer in 2023, and 16,710 will die of bladder cancer. Among these cases, approximately 75% of the patients diagnosed with bladder cancer will be found to have non-muscle-invasive bladder cancer (NMIBC) [1]. An analysis conducted using this dataset revealed noteworthy insights into cancer-specific survival (CSS) among individuals with NMIBC. Specifically, those with high-grade T1 staging and Tis exhibited a significant 19% risk of mortality attributed to bladder cancer. The study also uncovered that those with low-grade T1 and high-grade Ta staging also demonstrated a substantial 10% risk of cancer-specific mortality (CSM). These findings underscore the importance of recognizing the non-negligible risks associated with NMIBC, especially in cases involving specific staging characteristics [2].

Transurethral Resection of Bladder Tumor (TURBT) is considered the initial treatment and surgical management for non-muscle-invasive bladder cancer [3]. It is considered a crucial step in the management of NMIBC due to its ability to define patient risk class and prognosis; effectively determining the adjuvant therapies and follow-up schedules individualized for each patient [4]. The surgery is performed to remove all visible tumors and to provide specimens for pathological determination of staging and grade of the tumor. This examines whether the treatment using TURBT can be curative without additional therapies. The urologist will provide fractions of the exophytic part of the tumor, the underlying bladder wall, and the edges of the resection to the pathologist. Since there is a very thin distinction between superficial and invasive disease and different management, it is important to confirm the correct stage. Small specimens can be resected en bloc where the specimen contains the complete tumor plus a portion of the bladder wall. However, larger tumors should be resected in fractions with the underlying bladder wall and detrusor muscle (DM). The detrusor muscle being obtained has been considered a marker of resection quality and the absence of DM is associated with a higher risk of residual disease, especially in T1 tumors [5]. Pathology should be reported on each fraction to ensure the diagnosis is correct [6]. Commonly, TURBT has been carried out with subsequent Mitomycin C or gemcitabine therapy in low risk tumors to decrease the risk of recurrence [7].

The main objective of TURBT is a correct staging of the tumor, but a proper resection can also be therapeutic. For patients with low-risk disease, TUR with or without intravesical chemotherapy is the recommended treatment. In low-risk diseases, TURBT alone is shown to have a recurrence rate of 20% and a progression rate of 1% at 12 months, and TURBT with perioperative chemotherapy is found to have a recurrence rate of 10% and a progression rate of 1% at 12 months [8]. Since high-risk NMIBC tends to recur, it is recommended to perform a second restaging TURBT after an initial TUR confirms a high-risk tumor [9]. Performing multiple TURBTs is not a viable treatment option for patients with high-risk diseases (including CIS, any high-grade tumor, large tumors, and recurrent tumors), while patients may benefit from TURBT alone, the recommended treatment for the eradication of their disease includes Bacillus Calmette–Guérin (BCG) therapy and radical cystectomy [8,10].

Radical cystectomy stands as a recommended therapeutic option for individuals with NMIBC who exhibit inadequate responses to BCG therapy. In cases where BCG proves ineffective or the disease persists or progresses despite treatment, radical cystectomy, the surgical removal of the entire bladder, becomes a primary consideration. This intervention is particularly relevant for high-risk NMIBC cases, where the cancer has shown resistance to BCG immunotherapy. Radical cystectomy eliminates the existing tumor and serves as a definitive treatment, reducing the risk of disease recurrence and progression. Although the procedure involves significant lifestyle changes, due to the need to create a urinary diversion, it is often regarded as a curative option, especially when faced with BCG-unresponsive NMIBC. Lately, the adoption of an entirely intracorporeal robotic-assisted approach allows faster recovery with a shorter hospital stay, reduced surgical morbidity, and lower rates of complications.

First-line immune adjuvant therapy for intermediate and high-risk non-muscle-invasive bladder cancer is Bacillus Calmette–Guerin (BCG). BCG was developed from the *mycobacterium bovis* family in the early 20th century by Albert Calmette and Camille Guerin when developing a vaccine against tuberculosis. Berton Zbar later suggested the use of BCG to treat cancers, adopting BCG therapy to treat patients with melanoma and bladder cancer [11]. Trials performed by Zbar et al. demonstrated that inoculation of live tumor cells and BCG resulted in an inflammatory reaction to the BCG halting the progression of tumor growth [12]. In the 1970s, Morales et al. conducted a trial on 9 patients [13,14], leading to the approval of BCG as immunotherapy for NMIBC patients. The exact mechanism of action behind BCG therapy has not been elucidated, but it is thought that the immunologic response destroys bladder cancer cells and prevents tumor growth with variable effects on recurrence [14].

**Figure 1:**
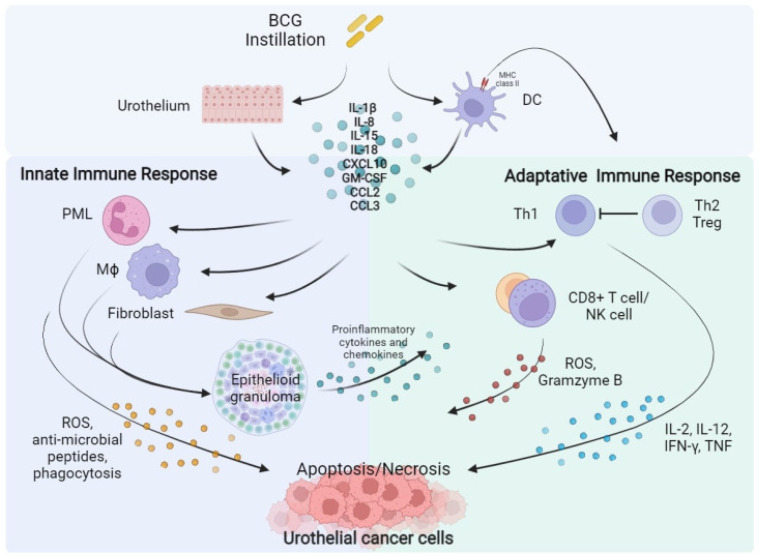
BCG mechanism of action to induce immune response [17].

The exact mechanism of action of BCG is unknown, but there are three ways in which BCG activates the immune system to kill malignant cells as shown in Figure 1. The first is by infection of urothelial cells with BCG. Fibronectin, a glycoprotein in the extracellular matrix, attaches BCG to tumor cells where it is then internalized. This leads to the second mechanism in which the innate immune system is activated, stimulating the activation of the reticuloendothelial system. This process activates granulocytes, macrophages, and T helper cells which secrete cytokines to recruit other immune cells to the bladder. The third way in which BCG activates the immune system is with antitumor activity through the adaptive immune system. BCG is presented to dendritic cells (DC) and then the DC presents them to other immune cells which will then produce apoptosis and necrosis-inducing agents. This is carried out with the use of both CD4+ and CD8+ cell activity as well as the use of natural killer cells against the tumor cells [15]. It was found that higher levels of leukocyturia resulted in a higher likelihood of success in reducing recurrence [16].

Although BCG is first-line therapy and considered safe to use, BCG has been shown to have many side effects that greatly affect treatment adherence. Studies show that over 70% of patients had some form of adverse effect with 8% of patients discontinuing BCG due to toxicity [18]. Many of these side effects present within a few hours of administration and are typically limited to 48–72 h [19]. Among the adverse effects are urinary frequency, cystitis, fever, and hematuria [20]. However, there are many side effects that are more severe. In the male urogenital system, prostatitis, specifically granulomatous prostatitis, has been observed in 41% of patients following BCG immunotherapy [21,22]. In addition, BCG therapy has been implicated as the cause of numerous cases of epididymo-orchitis [23]. Distant infections have also been observed as early complications of BCG dissemination, sometimes referred to as “BCGosis”. This infection has been observed to spread through the lymphatics or hematogenous pathways and can lead to granulomatous pneumonia, hepatitis, and dermatological, and ophthalmic manifestations. Additionally, cases of aortic BCG infection have been found resulting in aneurysm formation and potential rupture and death if left untreated [24]. Some patients, albeit very few, may develop severe, life-threatening sepsis with disseminated mycobacterial infection [20]. While some manifestations of BCG dissemination are clear, it can also present insidiously with symptoms of rash, arthritis, and pyrexia. Given the non-specific symptoms, a physician’s threshold of suspicion in those receiving treatment should be low, so these patients can be evaluated and treated with anti-tuberculosis medications if necessary [25].

Approximately one third of patients with NMIBC will not respond to BCG therapy and 50% of those with an initial response will experience recurrence or progression of their disease. The definition of BCG-unresponsive disease is based on the patient’s response to an initial course of BCG either by the presence of persistent or recurrent disease, evidence of tumor progression to a higher stage, or discontinuation of BCG due to intolerable side effects. Patients may be defined to be unresponsive after receiving adequate therapy which is defined as at least 5/6 induction instillations and at least 2/3 maintenance instillations for each cycle [26]. Currently, in the BCG-unresponsive stage both the EAU and AUA guidelines advocate for aggressive treatment with the standard of care being radical cystectomy with urinary diversion (with 10-year recurrence-free survival of 74–89%) [27]. Yet, much of the patient population for NMIBC are elderly, have other comorbidities, or are unwilling to undergo surgery due to the high perioperative morbidity and mortality associated with this treatment. High-risk elderly patients with NMIBC often necessitate aggressive treatment to prevent recurrence and progression to muscle-invasive disease. BCG therapy can be effective in these individuals; however, elderly patients may be more vulnerable and have lower tolerance to side effects, including local irritation, cystitis, and systemic symptoms, which can affect treatment adherence.

The recognition of patients who do not respond to BCG therapy or those with severe side effects that prevent further BCG instillation coupled with morbid surgical alternatives, and the increasing global shortage of BCG supply from 2012 when BCG production was forced to halt, that persisted through the COVID-19 pandemic, a renewed push for alternative therapies to BCG treatment is underway [28]. Pembrolizumab, which will be discussed later in this review, as a systemic therapy was explored and approved as a treatment possibility for patients with BCG-unresponsive carcinoma in situ (CIS) [29].

**Figure 2:**
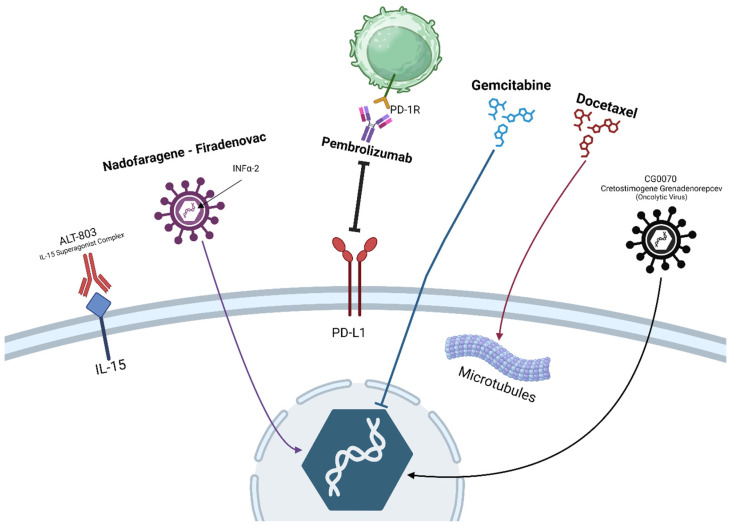
Current and future landscape of available treatments for non-muscle-invasive bladder cancer. Some therapies like BCG and MMC are not seen. All those shown are given through intravesical instillation (except pembrolizumab, which is given systemically). Drugs that are found in bold text are already approved for treatment; non-bolded drugs are under active investigation and have shown promising results (ALT-803, CG0070). Note: Created with BioRender.

In this review article, we seek to describe the current landscape of chemotherapeutics, immunotherapies, and gene therapies that have been recently approved (Figure 2) or are currently under investigation as BCG treatment alternatives or for the BCG-unresponsive state. Because, currently, multiple studies are ongoing, only published results will be presented.

## 2. BCG Alternative Therapies

### 2.1. BCG Combination Therapy with Interferon Alpha

For individuals who fail BCG monotherapy, combination therapy may demonstrate improved outcomes. As a combination therapy with BCG, interferon alpha2b demonstrated synergistic TH1 cell immuno-stimulation with varying degrees of success [30]. Some studies suggested that lower dose BCG in combination with interferon alpha led to lower levels of toxicity, while markedly increasing recurrence-free survival in BCG refractory patients. Other studies suggested that this treatment regimen was likely ineffective, though the reason for treatment failure was not elucidated [31,32,33]. A later Cochrane review found contradictory evidence on the use of interferon and BCG, ultimately concluding that there were no clear differences in recurrence or survival, additionally commenting that there was no evidence that discontinuing this therapy had any effect whatsoever [34]. Considering these data and recent advances in chemo and immunotherapy, BCG and interferon alpha combination therapy is not currently recommended and is infrequently used.

### 2.2. Intravesical Gemcitabine Monotherapy

Gemcitabine was originally designed as an antiviral drug in the 1980s but was also discovered to have a profound effect on leukemia cells. It acts as an antimetabolite chemotherapeutic, taking the place of deoxycytidine triphosphate in rapidly dividing cells and inhibiting DNA synthesis [35,36]. Due to this property, it has been used in many different types of cancers. As a bladder cancer therapeutic, it has been used intravesically for BCG-naive disease as well as BCG refractory disease. Currently, it is being tested in various combination therapies for high-risk and refractory disease with docetaxel, as well as with cabazitaxel and cisplatin [37,38]. Currently, the comparison of gemcitabine monotherapy to BCG is of low evidence [39]. Some studies comparing the two were seen to have similar rates of recurrence (BCG 30% and gemcitabine 25%) and progression (BCG 37.5% and gemcitabine 33%) [40]. The efficacy of intravesical gemcitabine and intravesical BCG were almost equal in the treatment of NMIBC [38]. In another study testing the efficacy of gemcitabine, Addeo et al. found a recurrence-free survival (RFS) of 72% in a 36-month follow-up [41]. In terms of its safety profile, gemcitabine demonstrates good tolerability and minimal toxicity up to 2000 mg/50 mL saline for 2 h instillations [42]. Gemcitabine, like many of the chemotherapeutics discussed, has a relatively high molecular mass, limiting the amount of systemic absorption. The adverse events most observed were pyrexia and body aches. Common local adverse events include those related to chemical cystitis such as urgency, dysuria, hematuria, and suprapubic discomfort [38,42,43]. Compared to BCG therapy, dysuria, and urinary frequency were seen to be common side effects between the two; however, rates of these adverse effects were much greater in those with BCG therapy [38,40]. Between gemcitabine and BCG therapy, it is found that they are very similar in treatment efficacy with gemcitabine presenting with lower risk for adverse effects. Future directions for gemcitabine monotherapy involve incorporating TAR-200, a low-dose gemcitabine delivery system that works via osmotic gradient-dependent release over a 21-day period, combined with cetrelimab (anti-PD1 antibody), which is given systemically, the initial preliminary results from phase 2 of the study (SunRISe-1) are encouraging. Yet, official results are pending [44].

#### Intravesical Sequential Gemcitabine/Docetaxel

Recently, there has been growing evidence to support the role of sequential Gem/Doce instillations as an alternative to BCG therapy, and the need to pursue this path of treatment stemmed from the BCG shortage, as previously discussed. Docetaxel (Doce) in combination with Gemcitabine (Gem) showed promising results in different disease risk groups. The mechanism of action of Gem/Doce is determined by both individual parts. Docetaxel works by disrupting cell division by inhibiting microtubules while Gemcitabine works by disrupting DNA synthesis as a deoxycytidine nucleoside analog, as mentioned before [45]. The combination is given once weekly for six weeks, which is similar to BCG therapy. The regiment has an induction course followed by maintenance therapy that is given once monthly for at least one year [46,47]. Currently, there is a lack of standardization for administration protocols and duration of treatment. The commonly used dosage for gemcitabine is 1 g; however, a few studies also described using 2 g of gemcitabine. For docetaxel, the commonly used dosage is 37.5 mg [46,47,48]. The sequential treatment is administrated intravesically by a catheter, starting with gemcitabine; the patient will hold the drug for an hour, followed by bladder drainage and the administration of docetaxel, the agent is held for another hour, and then the patient is instructed to void. McElree et al. demonstrated that in BCG-naïve high-risk NMIBC, recurrence rates between sequential Gem/Doce and BCG were similar [47]. The same group published a subsequent larger cohort and demonstrated that the Gem/Doce combination had better RFS for any recurrence (HR 0.56, *p* = 0.02) and high-grade recurrences compared to BCG (HR 0.57, *p* = 0.04) [48]. A subsequent multicenter study analyzed Gem/Doce in comparison to BCG in the intermediate-risk NMIBC. RFS in a median follow-up time of 48.6 months was similar between the groups; in this context, it is worth mentioning that the majority of the patients had TaLG disease, either primary or recurrent, and not TaHG disease [46]. The maintenance regimen in the Gem/Doce combination involved a monthly administration of the drug over a period of a year; as this regimen has not been standardized yet, a longer maintenance (2–3 years) period may show additional benefit. The two monotherapies are well tolerated. For the BCG-unresponsive setting, Yim et al. retrospectively analyzed 102 patients who received Gem/Doce induction and maintenance therapy for up to 2 years, and their 12- and 24-month RFS was 65% and 49%, respectively [49]. The adverse effects profile is similar to those of gemcitabine alone and has a favorable side effect profile over BCG therapy. McElree reported that patients who received Gem/Doce were less likely to discontinue treatment compared to BCG (2.9% vs. 9.2% *p* = 0.02). In their analysis, bladder spasms occurred most commonly with Gem/Doce therapy (21%), followed by UTI (8.7%) and urgency and frequency (8.7%). Moreover, the side effect profile of the BCG arm demonstrated that 49.4% of the patients had adverse events with urgency (11.5%), dysuria (12.6%), hematuria (10.9%), and UTI (6.9%), and 3 events of grade 3 or above, one resulting in mortality. In the study by McElree et al., oxybutynin was given prophylactically to patients treated with Gem/Doce [48]. The BRIDGE trial, a phase 3 prospective trial investigating Gem/Doce compared to BCG instillations in high-risk NMIBC is currently accruing patients [50].

### 2.3. Intravesical Mitomycin C Therapy

Mitomycin C (MMC) was first described as an antibiotic in the 1950s by Japanese microbiologists after being isolated from the bacterium *Streptomyces caespitosus* [51]. It was noted to have antitumor activity through alkylation of DNA [51,52]. Because of its alkylating properties, MMC’s role as an antibiotic has been limited, though it has flourished as a chemotherapeutic agent including in bladder cancer [53]. The primary role of MMC in the treatment of NMIBC is as an adjuvant intravesical therapy for low to intermediate-risk tumors immediately following TURBT or as 6 induction instillations. Many studies have shown a recurrence benefit when using MMC in addition to TURBT when compared to TURBT alone (MMC + TURBT 44.8% and TURBT alone 58.8%, *p* < 0.001) [7]. Treatment with MMC is highly non-uniform and response is variable due to differing acceptable doses ranging from 20 to 60 mg with different instillation durations (between 0.5 and 2 h) when given up to 6 h following TURBT, with increased effectiveness of treatment if the first instillation is given within 2 h following resection [7,54,55]. The adjuvant schedule for MMC treatment typically consists of an induction phase, which usually begins after the TURBT where MMC is instilled into the bladder once a week for six consecutive weeks. In high-risk cases, a second phase, a maintenance phase, is considered, which involves MMC instillations at regular intervals for an extended period. However, when comparing the efficacy of BCG and MMC in preventing progression and recurrence, several studies have concluded that BCG is more effective [7,56,57]. The most common treatment approach using MMC is post-TURBT instillation of chemotherapy. For low-risk NMIBC, this can be considered complete treatment [58,59]; however, intermediate and high-risk NMIBC often necessitate treatment with maintenance therapy as well, similar to BCG therapy. A Cochrane review assessed BCG vs. MMC covering 12 RCTs and 2932 participants in intermediate and high-risk NMIBC, in the analysis of BCG to MMC no difference was found in overall survival (HR 0.97, CI 95% 0.79–1.2), progression-free survival (HR 0.96, 95% CI 0.73–1.26) or recurrence-free survival (HR 0.88, 95% CI 0.71–1.09) between the groups. Overall, the level of evidence is low due to patients’ selection bias and performance bias. However, more events of serious adverse effects were observed in the BCG group vs. the MMC group (pooled RR of 2.31 95% CI 0.82–6.52) [60].

Although there is little evidence of a difference between MMC and BCG, MMC has been considered a safe and effective treatment for low to intermediate-risk NMIBC, given the fact that MMC has high molecular mass leading to very low systemic absorption and toxicity [60]. Studies analyzing MMC alone noted common side effects of exanthema in 5.4% of patients and irritative urinary symptoms in 5% of patients [58,61]. As well, adverse events of dysuria, urinary frequency, urgency, as well as hematuria, and chemical cystitis were also commonly noted [62]. Because treatment with MMC often involves immediate post-TURBT intravesical instillation, there is a risk of bladder perforation, which can have devastating consequences due to drug extravasation and subsequent chemical injury [62,63,64]. Much of the commonly described toxicities of MMC are local, with chemical reactions of the skin, delayed-type hypersensitivity, or irritation of the bladder being the most common [65]. The classic systemic toxicity associated with MMC is myelosuppression, usually manifesting within the first week after treatment. Other adverse effects of MMC therapy included urinary frequency, incontinence, cramps, prostatitis, fever, general malaise/discomfort, fatigue, allergic reactions, dysuria, skin alterations, and cystitis, including bacterial cystitis and drug-induced cystitis [60].

#### Thermo-Chemotherapy 

Another modality for BCG-unresponsive patients works to increase the efficacy of chemotherapy. Various methods have been investigated, including the use of devices to create a more favorable environment for tumor destruction. One of the common types of therapy is known as chemo-hyperthermia(C-HT), originally known as the radiofrequency-induced thermo-chemotherapy effect (RITE). Historically, RITE was used in NMIBC [65], but advances in technology and understanding have led to a newer form of this therapy called hyperthermic intravesical chemotherapy (HIVEC). Both modalities utilize the same underlying principle of hyperthermia. How chemo-hyperthermia is used is generally either as a neoadjuvant before TURBT, as an adjuvant or prophylactic or ablative form of treatment. While there are many different devices and protocols utilized in this form of treatment, the principle is to increase the environment temperature to 43 °C during intravesical instillation of chemotherapy, most commonly Mitomycin C (MMC) [66,67]. This supra-physiologic temperature has been observed to increase tumor perfusion and change cell membrane characteristics to increase the intracellular concentration of chemotherapeutics [68]. The schedule utilized for adjuvant therapy is similar to MMC alone, with an induction cycle and weekly maintenance for 6 weeks, depending on the regiment [69]. Colombo and colleagues explored this in a 2011 randomized controlled trial investigating the differences between MMC vs. chemo-hyperthermia, finding that over 24 months, the recurrence rate in MMC patients was significantly higher than those treated with chemo-hyperthermia, with a 59% relative reduction in NMIBC when chemo-hyperthermia is used compared with MMC alone. Observational trials have examined progression-free survival in patients undergoing HIVEC, some have noted better progression outcomes compared to BCG whereas others have noted no significant difference [69]. Plata et al. examined a single-arm study that found that RFS for HIVEC treatment in the intermediate risk and high risk groups at 1 year were 86.7% and 80.3%, respectively, and no difference was found between the groups [70]. As research on the subject has spanned many decades, data on adverse events is difficult to compare due to the use of currently invalidated questionnaires. However, a 2011 systematic review quantified some of the adverse events reported by previous studies. The most common adverse events reported during treatment were bladder spasms and pain occurring in 21.6% and 17.5% of patients, respectively. Post-treatment, the most commonly reported symptoms were mild lower urinary tract symptoms of dysuria, urgency, nocturia, and frequency, as well as hematuria [71]. In a 2021 study investigating the adverse effects of HIVEC in patients who failed BCG or had contraindications, it was found that the most common adverse effects were dysuria, frequency, and incontinence [72]. This study also explored symptoms related to each instillation and noted additional systemic adverse effects. As with mitomycin C alone, myelosuppression is a possible adverse effect if the mitomycin C is systemically absorbed; however, the risk of this effect is negligible [73]. In total, 8.1% of those studied experienced mild flu-like symptoms, 7.3% had abdominal pain, and 5.7% had nausea. Multiple studies have also reported nonspecific exanthem, similar in incidence to those reported after MMC alone, which appears in up to 6% of patients. Specific to certain types of HIVEC is a risk for a posterior wall thermal reaction, in which there is hyperemia at the position of contact between the device and the bladder wall. These events were often mild and resolved over months [74,75]. There is also a risk for bladder contraction, which is also present in MMC therapy alone. However, studies have not concluded that this was exclusively due to the hyperthermic component of treatment [71]. In sum, many of the common adverse effects associated with HIVEC were considered mild and tolerable by the majority of patients. 

### 2.4. Pembrolizumab

In 2021, the KEYNOTE-057 study was published, which led to the approval of pembrolizumab by the FDA for the treatment of BCG-unresponsive disease. Pembrolizumab was the first alternative to radical cystectomy in patients with BCG-unresponsive CIS disease. Pembrolizumab is a PD-1 inhibitor that has been approved as second-line therapy for metastatic urothelial carcinoma and as first-line for cisplatin-ineligible patients. It is known that the PD1/PD-L1 pathway activation is seen in those with resistance to BCG with NMIBC, and PD-L1 expression was seen in tumors that relapsed after BCG treatment. Thus, it was hypothesized that the inhibition of this pathway would improve outcomes. Pembrolizumab is administrated intravenously every 3 weeks for up to 24 months at a dose of 200 mg. Two cohorts were studied, the first: cohort A examined the role of monotherapy with systemic pembrolizumab in patients with CIS in the BCG-unresponsive state; at 3 months, 41% (39 of 96 patients) had a complete response (CR) and, of those, 46% (18 patients) had a CR at 12 months. Complete response was defined in this study as the absence of high-risk NMIBC or progressive disease. Adverse events happened in 66% of the patients and included diarrhea, fatigue, and pruritis, 7% of the patients developed hypothyroidism, and 5% developed hyperthyroidism. Grade 3 or above AE occurred in 13 patients (13%), which included hyponatremia, arthralgia, and pruritis. The trial found that pembrolizumab was able to achieve a 40% response rate, which exceeds that observed in other intravesical therapies [29]. Most recently, preliminary results from cohort B of the phase 2 Keynote-057 have been published. Cohort B consists of patients with papillary tumors without CIS, with results measured by disease-free survival (DFS), which is commonly used to determine outcomes of treatment for papillary tumors. These encouraging initial results showed a disease-free survival (DFS) rate of 43.5% for high-risk NMIBC at 12 months (95% CI, 34.9–51.9) [76]. This showed promising preliminary results; however, official results are pending.

### 2.5. Novel Therapies

While many studies are being performed on known therapies, NMIBC research is also directed at advances in immunotherapy. Many new immunotherapy options are being researched and tested in search of an efficient and effective alternative to BCG therapy.

#### 2.5.1. ALT-803

ALT-803/N-803 is one of the immunotherapies currently being tested. The IL-15 receptor is crucial for the recruitment and activation of NK cells and CD8+ T cells. ALT-803 is evidenced to be an IL-15 superagonist with a longer half-life than recombinant IL-15 that results in synergistic immune system activation of NK and T cells, shown to have a 71% CR rate, and a 48% EFS rate thus far with a longer half-life than recombinant IL-15 [77,78]. In a phase 1 trial, all 9 patients treated with a combination of BCG and ALT-803 were found to be disease-free at 24 months after treatment with no severe adverse events. Phase 2 trials are now being conducted, as well as a phase 2/3 trial (QUILT-3.032) testing BCG and ALT-803 in BCG-unresponsive patients. Preliminary results show that, in a median follow-up time of 26.6 months, 96% of patients with CIS did not progress to MIBC, and patients with papillary disease achieved a DFS of 57% and 48% at 12 and 24 months, respectively [79]. However, the latest FDA update (May 2023) declined to approve the treatment before further safety issues are resolved.

#### 2.5.2. Oncolytic Viral Delivery System

Other novel therapies being studied include viral delivery systems for gene therapy for therapeutic benefit in the treatment of BCG-unresponsive NMIBC. Two promising viral delivery systems include Nadofaragene-Firadenovac (Adstiladrin) and CG0070. 

##### Nadofaragene-Firadenovac

Nadofaragene-Firadenovac (Adstiladrin), also known as rAd-IFNa/Syn3, is one such delivery system that uses a recombinant adenovirus in conjunction with a polyamide surfactant to deliver a copy of human interferon alpha-2b cDNA, which will enhance the viral transduction of the urothelium resulting in local interferon alpha-2b production [80]. Interferon alpha-2b has previously been shown to promote immune response and has been used in preventing the recurrence of NMIBC in intermediate and high-risk diseases after treatment with BCG [34,80,81]. In clinical trials, interferon alpha demonstrated the ability to elicit complete responses without reaching the maximum tolerated dose. While an anti-tumor effect has been demonstrated, current trials have not investigated doses higher than 100 MU. Currently, Adstiladrin is administered once every 3 months for up to 12 months (4 vials) through a catheter at a dose of 75 mL (3 × 10 [11] of viral particles per mL), the patient is told to hold their urine for an hour. In the various trials that have been performed, efficacy was demonstrated to be limited both in the role of adjuvant maintenance therapy, as well as in the role of immediate post-operative instillation [81]. 

Boorjian et al. demonstrated in a single-arm phase 3 study that, in a cohort of CIS patients, BCG-unresponsive patients were seen to have CR in 53.4% at 3 months and 24.3% at 12 months. In this study, complete response was defined as a negative urine cytology and cystoscopy assessed by the physician. In the high-grade Ta or T1 cohort, high-grade RFS at 3 and 12 months was 72.9% and 43.8%, respectively. Recurrence-free survival if the patient was alive and without recurrence of high-grade disease or progression. AEs were reported in 70.1% of patients, with 3.8% ≥ G3 AEs and 1.9% electing to discontinue treatment due to AEs. The common AEs reported were transient, lasting on average two days and consisting of lower urinary tract symptoms which were treatable with anticholinergics, hyperglycemia, hypertriglyceridemia, installation site discharge, headache, fatigue, and nausea [80]. Following this study, Adstiladrin was the first novel intravesical drug to be approved by the FDA after BCG in December 2022.

##### CG0070

The other viral delivery system, CG0070 (Cretostimogene Grenadenorepvec), is a targeted oncolytic virus given intravesically. The delivered cargo system induces its immune response through stimulation of GM-CSF in Rb protein-deficient cells. GM-CSF expression typically leads to downstream granulocyte, macrophage, lymphocyte, and dendritic cell activation, which has been implicated in antitumor activity in many malignancies [82,83,84]. The phase 1 CG0070 monotherapy trial showed the ability to achieve sustained concentrations in patient urine when administered intravesically. Subsequent administrations lead to lower urinary concentrations, likely due to the development of anti-adenoviral antibodies [85]. Median CR was 48.6% with a median duration of 10.4 months with common AEs of lower urinary tract symptoms, fatigue, arthralgia, myalgia, bladder discomfort, abdominal pain, and influenza-like illnesses appearing most commonly [86]. The subsequent phase 2 trial showed an overall 6-month CR of 47% for all patients with similar tolerable toxicities [87]. Combination therapy of pembrolizumab and CG0070 was also evaluated, with phase 2 trials showing 92% achieving CR at 3 months, 88% of those at 6 months, 82% of those at 9 months, and 75% at 12 months [88]. At this time, the phase 3 BOND-003 trial is being performed, which will further investigate CG0070 monotherapy.

Both adenoviral delivery systems (CG0070 and Nadofaragene-Firadenovac) discussed in this section have shown that they can concentrate in urine to a greater extent than previously demonstrated with their base therapeutic, likely lending to their CR. This, combined with relatively tolerable AEs, demonstrates the potential of these therapeutics in the treatment of NMIBC, pending the completion of the phase 3 trial (BOND-003, NCT04452591) for CG0070 and the persistent safety of NF.

## 3. Discussion

In 2012, one of the major manufacturers of BCG was forced to halt production due to inconsistencies, leading to a shortage of the therapeutic agent that was projected to last until 2020. As of the writing of this review, the availability of BCG is still limited, making it necessary to revisit old therapeutics while searching for new ones.

The current first-line therapy for NMIBC is TURBT followed by BCG induction and maintenance. Intravesical chemotherapy exists as an alternative to BCG and while other options exist, comparative studies are relatively small, but when present they show the superiority of BCG. Chemotherapeutics have a varied rate of complete response ranging from 18 to 50% with an EFS of 4–60%, so while there are options for alternative therapy in this pharmaceutic space, it is clear there is a necessity for further studies and alternatives [89].

The current promising alternative to BCG therapy is sequential Gem/Doce chemotherapy that demonstrated efficacy in the BCG naïve high-risk NMIBC, recurrence rates between sequential Gem/Doce and BCG were similar. A subsequent larger cohort study that compared the Gem/Doce combination to BCG showed superior RFS for any recurrence (HR 0.56, *p* = 0.02) and high-grade recurrences (HR 0.57, *p* = 0.04) for the Gem/Doce combination, the side effect profile seem to be better tolerated than BCG therapy, with lower rates of treatment termination. Common side effects include bladder spasms and nausea, and as implied from the combination, there is no risk for BCG-itis. Yet, as the current data on sequential Gem/Doce administration is retrospective and mostly stems from one center, there is a lack of protocol standardization both for the duration and dosage of the agents. A phase 3 trial comparing the effectiveness and prevention of recurrence of high-grade NMIBC in BCG-naive patients treated with Intravesical Gem/Doce combination chemotherapy and BCG monotherapy (the BRIDGE trial) is currently accruing. Its results may better standardize the administration protocol schedule and dosage used as well as provide level 1 comparative data of Gem/Doce to BCG.

In the BCG-unresponsive NMIBC stage, systemic pembrolizumab treatment has achieved a complete response rate of 19% at 1 year in the BCG-unresponsive stage for patients with CIS (cohort A). While this may seem like a low rate, these patients were spared from radical cystectomy. Yet, with the expense of a 13% rate of serious complications (grades 3 and above). Preliminary cohort B results (papillary tumors without CIS) were favorable, but official results are pending. However, currently, as pembrolizumab is administrated systemically, it may lead to urological departments diverting its administration to medical oncologists due to logistics associated with intravenous administration and because medical oncologists are more familiar with the adverse effects profile. Nadofaragene-Firadenovac has been recently approved by the FDA for the treatment of unresponsive bladder cancer. The CR rate at 1 year approached 25% with a relatively low rate of serious complications (3.8%). Furthermore, the intravesical route of administration may be logistically easier for administration by urologists than pembrolizumab. However, for both new drugs, the long-term efficacy has yet to be published. Consequenstly, many centers may still recommend early radical cystectomy with a 5-year cancer-specific survival rate exceeding 90% [90].

In the last few years, examining the effect of novel therapies and delivery systems such as N-803, CG0070, TAR-200 delivery system [44], and TARA-002 intravesical instillation of low-virulence Streptococcus pyogenes preparation [91] has demonstrated promising preliminary results, and they may become alternative therapies in the BCG-unresponsive state or in places where there is a BCG shortage. However, while the future of the treatment possibilities is brighter than ever before, official results that have been published remain undecided if these treatments can be a viable option in the BCG-unresponsive state or as an alternative to BCG in earlier stages of the disease, and more research on the matter must be conducted.

Future directions for research on this topic can address points of concern that were noted as we were compiling primary sources. Although there is a wide variety of chemotherapeutics, immunotherapeutics, and gene therapies to treat NMIBC, many studies compare alternative therapeutics to BCG and not to each other, which may be another modality to determine efficacy and best options for patients. The summaries and findings here, which can be found in Table 1, may be further substantiated and enhanced through additional head-to-head studies and randomized control trials comparing therapies to one another.

There are several limitations to this review. Only studies indexed online in repositories such as PubMed^®^ and Google Scholar, and the results of some ongoing clinical trials were utilized in our final review leading to the possibility that studies that were not indexed were omitted from the literature search. There are also multiple ongoing studies investigating BCG alternative treatment that were omitted, as complete data is currently not available; therefore, this review is not exhaustive of all therapies that are being studied today. As the purpose of this review was to describe the current landscape of therapeutics, these limitations should not call into question the value of this review as a scholarly resource. While we do believe this review to be coherent and promising for alternative treatment options for patients with NMIBC, this study should not be used to guide clinical decisions, as some treatments listed have limited practical application to patient care due to high costs, current experimental nature, or lack of availability.

## 4. Conclusions

The landscape of non-invasive bladder cancer treatment is complex. Since the introduction of BCG in the 1970s, slow progress has been made in developing alternative treatments. Recently, novel therapies have been and are being approved for the treatment of NMIBC specifically in the BCG-unresponsive stage (pembrolizumab and Nadofaragene-Firadenovac), sparing patients from radical cystectomy. In the earlier stage of the disease, Gem/Doce is gaining ground for a viable alternative to BCG therapy. However, robust data have yet to be published, and as we advance in trying to spare “bladders”, it is prudent to develop reliable biomarkers to detect signs of progression, as regional or metastatic disease may develop even in the NMIBC stage.

This paper will facilitate further work in the study of NMIBC treatment by providing a concise and up-to-date review of BCG and currently available alternative treatments, which can allow future researchers a place to begin their own work. A point of inquiry is that although there is a wide variety of chemotherapeutics, immunotherapeutics, and gene therapies to treat NMIBC, many of the studies performed are those comparing alternative therapeutics to BCG and not to each other. The summaries and findings here may be further substantiated and enhanced through additional randomized controlled trials comparing therapeutics to one another.

## Figures and Tables

**Table 1:** Relevant outcomes for alternatives to BCG therapy in BCG-responsive and unresponsive patients.

Study	Disease Grade	Treatment	*n*	Follow-Up	CR	RFS	Progression Risk
Plata et al [69]	Intermediate and High	Thermo-chemotherapy	IR: 297 HR: 205	12 months	---	IR: 86.7% HR: 80.3%	IR: 2.2% HR: 6.1%
Tan et al [45]	Intermediate	Gem/Doce	82	24 months	---	75% (85% at 12 months)	---
McElree et al [46]	High	Gem/Doce	107	24 months	---	84%	---
Addeo et al [40]	BCG-unresponsive	Gemcitabine	54	36 months	---	72%	11.1%
Yim et al [48]	BCG-unresponsive	Gem/Doce	102	24 months	---	49% (65% at 12 months)	2.9%
Addeo et al [40]	BCG-unresponsive	Mitomycin C	55	36 months	---	61%	18.2%
Balar et al [28]	BCG-unresponsive	Pembrolizumab (cohort A) KEYNOTE057	96	12 months	18.8%	---	---
Necchi et al [75]	BCG-unresponsive	Pembrolizumab (cohort B) KEYNOTE057	132	12 months	---	43.5%	---
Chamie et al [78]	BCG-unresponsive	ALT-803 (cohort A, CIS)	83	24 months	71.0%	---	4%
Chamie et al [78]	BCG-unresponsive	ALT-803 (cohort B, papillary)	77	24 months	---	48%	---
Boorjian et al [79]	BCG-unresponsive	Nadofaragene-Firadenovac (cohort A, CIS)	103	12 months	24.3%	---	2.9%
Boorjian et al [79]	BCG-unresponsive	Nadofaragene-Firadenovac (cohort B, HG Ta or T1)	48	12 months	---	43.8%	6.2%
Packiam et al [86]	BCG-unresponsive	CG0070	45	6 months	47%	---	2.2%

IR—Intermediate Risk; HR—High Risk; *n*—Number of Patients; RFS—Recurrence-free Survival; CR—Complete Response; BCG—Bacillus Calmette–Guerin; Gem/Doce—Gemcitabine and Docetaxel combination therapy.

## References

[1] Siegel R.L., Miller K.D., Wagle N.S., Jemal A. (2023). Cancer statistics, 2023. CA Cancer J. Clin..

[2] Ślusarczyk A., Zapała P., Zapała Ł., Borkowski T., Radziszewski P. (2023). Cancer-Specific Survival of Patients with Non-Muscle-Invasive Bladder Cancer: A Population-Based Analysis. Ann. Surg. Oncol..

[3] Osman Y., Elawdy M., Taha D.-E., Zahran M.H., Abouelkheir R.T., Sharaf D.E., Mosbah A., Dein B.A.-E. (2023). Bladder perforation as a complication of transurethral resection of bladder tumors: The predictors, management, and its impact in a series of 1570 at a tertiary urology institute. Int. Urol. Nephrol..

[4] Gallioli A., Diana P., Fontana M., Territo A., Rodriguez-Faba Ó., Gaya J.M., Sanguedolce F., Huguet J., Mercade A., Piana A. (2022). En Bloc Versus Conventional Transurethral Resection of Bladder Tumors: A Single-center Prospective Randomized Noninferiority Trial. Eur. Urol. Oncol..

[5] Mastroianni R., Brassetti A., Krajewski W., Zdrojowy R., Al Salhi Y., Anceschi U., Bove A.M., Carbone A., De Nunzio C., Fuschi A. (2021). Assessing the Impact of the Absence of Detrusor Muscle in Ta Low-grade Urothelial Carcinoma of the Bladder on Recurrence-free Survival. Eur. Urol. Focus.

[6] Anastasiadis A., de Reijke T.M. (2012). Best practice in the treatment of nonmuscle invasive bladder cancer. Ther. Adv. Urol..

[7] Sylvester R.J., Oosterlinck W., Holmang S., Sydes M.R., Birtle A., Gudjonsson S., De Nunzio C., Okamura K., Kaasinen E., Solsona E. (2016). Systematic Review and Individual Patient Data Meta-analysis of Randomized Trials Comparing a Single Immediate Instillation of Chemotherapy After Transurethral Resection with Transurethral Resection Alone in Patients with Stage pTa–pT1 Urothelial Carcinoma of the Bladder: Which Patients Benefit from the Instillation?. Eur. Urol..

[8] Matulewicz R.S., Steinberg G.D. (2020). Non-Muscle-invasive Bladder Cancer: Overview and Contemporary Treatment Landscape of Neoadjuvant Chemoablative Therapies. Rev. Urol..

[9] Ramírez-Backhaus M., Domínguez-Escrig J., Collado A., Rubio-Briones J., Solsona E. (2012). Restaging transurethral resection of bladder tumor for high-risk stage Ta and T1 bladder cancer. Curr. Urol. Rep..

[10] Lamm D.L., Thor D.E., Stogdill V.D., Radwin H.M. (1982). Bladder cancer immunotherapy. J. Urol..

[11] Mukherjee N., Wheeler K.M., Svatek R.S. (2019). Bacillus Calmette-Guérin (BCG) treatment of bladder cancer: A systematic review and commentary on recent publications. Curr. Opin. Urol..

[12] Zbar B., Bernstein I., Tanaka T., Rapp H.J. (1970). Tumor immunity produced by the intradermal inoculation of living tumor cells and living Mycobacterium bovis (strain BCG). Science.

[13] Morales A., Eidinger D., Bruce A.W. (2017). Intracavitary Bacillus Calmette-Guerin in the Treatment of Superficial Bladder Tumors. J. Urol..

[14] Guallar-Garrido S., Julián E. (2020). Bacillus Calmette-Guérin (BCG) Therapy for Bladder Cancer: An Update. Immunotargets Ther..

[15] Fuge O., Vasdev N., Allchorne P., Green J.S. (2015). Immunotherapy for bladder cancer. Res. Rep. Urol..

[16] Saint F., Patard J.J., Irani J., Salomon L., Hoznek A., Legrand P., Debois H., Abbou C.C., Chopin D.K. (2001). Leukocyturia as a predictor of tolerance and efficacy of intravesical BCG maintenance therapy for superficial bladder cancer. Urology.

[17] Audisio A., Buttigliero C., Delcuratolo M.D., Parlagreco E., Audisio M., Ungaro A., Di Stefano R.F., Di Prima L., Turco F., Tucci M. (2022). New Perspectives in the Medical Treatment of Non-Muscle-Invasive Bladder Cancer: Immune Checkpoint Inhibitors and Beyond. Cells.

[18] Brausi M., Oddens J., Sylvester R., Bono A., van de Beek C., van Andel G., Gontero P., Turkeri L., Marreaud S., Collette S. (2014). Side effects of Bacillus Calmette-Guérin (BCG) in the treatment of intermediate- and high-risk Ta, T1 papillary carcinoma of the bladder: Results of the EORTC genito-urinary cancers group randomised phase 3 study comparing one-third dose with full dose and 1 year with 3 years of maintenance BCG. Eur. Urol..

[19] Koch G.E., Smelser W.W., Chang S.S. (2021). Side Effects of Intravesical BCG and Chemotherapy for Bladder Cancer: What They Are and How to Manage Them. Urology.

[20] Waked R., Choucair J., Chehata N., Haddad E., Saliba G. (2020). Intravesical Bacillus Calmette-Guérin (BCG) treatment’s severe complications: A single institution review of incidence, presentation and treatment outcome. J. Clin. Tuberc. Other Mycobact. Dis..

[21] Oates R.D., Stilmant M.M., Freedlund M.C., Siroky M.B. (1988). Granulomatous prostatitis following bacillus Calmette-Guerin immunotherapy of bladder cancer. J. Urol..

[22] Logan J.K.B., Walton-Diaz A., Rais-Bahrami S., Merino M.J., Turkbey B., Choyke P.L., Pinto P.A. (2014). Changes observed in multiparametric prostate magnetic resonance imaging characteristics correlate with histopathological development of chronic granulomatous prostatitis after intravesical Bacillus Calmette-Guerin therapy. J. Comput. Assist. Tomogr..

[23] Harada H., Seki M., Shinojima H., Miura M., Hirano T., Togashi M. (2006). Epididymo-orchitis caused by intravesically instillated bacillus Calmette-Guérin: Genetically proven using a multiplex polymerase chain reaction method. Int. J. Urol..

[24] Haddad J., Chalret du Rieu H., Ducasse E., Berard X., Caradu C. (2023). BCG Aortitis, a Rare Complication of BCG Therapy. EJVES Vasc. Forum..

[25] Oliveira A.A., Morais J., Ribeiro J., Gouveia P.F. (2021). Systemic infection following intravesical therapy with BCG. BMJ Case Rep..

[26] Non-Muscle-Invasive Bladder Cancer—DISEASE MANAGEMENT—Uroweb. https://uroweb.org/guidelines/non-muscle-invasive-bladder-cancer/chapter/disease-management.

[27] Lebacle C., Loriot Y., Irani J. (2021). BCG-unresponsive high-grade non-muscle invasive bladder cancer: What does the practicing urologist need to know?. World J. Urol..

[28] Veeratterapillay R., Heer R., Johnson M.I., Persad R., Bach C. (2016). High-Risk Non-Muscle-Invasive Bladder Cancer—Therapy Options During Intravesical BCG Shortage. Curr. Urol. Rep..

[29] Balar A.V., Kamat A.M., Kulkarni G.S., Uchio E.M., Boormans J.L., Roumiguié M., Krieger L.E.M., Singer E.A., Bajorin D.F., Grivas P. (2021). Pembrolizumab monotherapy for the treatment of high-risk non-muscle-invasive bladder cancer unresponsive to BCG (KEYNOTE-057): An open-label, single-arm, multicentre, phase 2 study. Lancet Oncol..

[30] Luo Y., Chen X., Downs T.M., DeWolf W.C., O’Donnell M.A. (1999). IFN-α 2B Enhances Th1 Cytokine Responses in Bladder Cancer Patients Receiving Mycobacterium bovis Bacillus Calmette-Guérin Immunotherapy. J. Immunol..

[31] O’Regan T., Tatton M., Lyon M., Masters J. (2015). The effectiveness of BCG and interferon against non-muscle invasive bladder cancer: A New Zealand perspective. BJU Int..

[32] O’Donnell M.A., Krohn J., Dewolf W.C. (2001). Salvage intravesical therapy with interferon-alpha 2b plus low dose bacillus Calmette-Guerin is effective in patients with superficial bladder cancer in whom bacillus Calmette-Guerin alone previously failed. J. Urol..

[33] Prasad S.M., Eyre S., Loughlin K.R. (2013). Salvage combination intravesical immunotherapy with Bacillus Calmette-Guérin and interferon-α2B: Impact on recurrence, progression, and survival. Hosp. Pract..

[34] Shepherd A.R.H., Shepherd E., Brook N.R. (2017). Intravesical Bacillus Calmette-Guérin with interferon-alpha versus intravesical Bacillus Calmette-Guérin for treating non-muscle-invasive bladder cancer. Cochrane Database Syst. Rev..

[35] Lee K., Kim D.-E., Jang K.S., Kim S.J., Cho S., Kim C. (2017). Gemcitabine, a broad-spectrum antiviral drug, suppresses enterovirus infections through innate immunity induced by the inhibition of pyrimidine biosynthesis and nucleotide depletion. Oncotarget.

[36] Ciccolini J., Serdjebi C., Peters G.J., Giovannetti E. (2016). Pharmacokinetics and pharmacogenetics of Gemcitabine as a mainstay in adult and pediatric oncology: An EORTC-PAMM perspective. Cancer Chemother. Pharmacol..

[37] DeCastro G.J., Sui W., Pak J.S., Lee S.M., Holder D., Kates M.M., Virk R.K., Drake C.G., Anderson C.B., James B. (2020). A Phase I Trial of Intravesical Cabazitaxel, Gemcitabine and Cisplatin for the Treatment of Nonmuscle Invasive bacillus Calmette-Guérin Unresponsive or Recurrent/Relapsing Urothelial Carcinoma of the Bladder. J. Urol..

[38] Djafari A.A., Javanmard B., Razzaghi M., Hojjati S.A., Razzaghi Z., Faraji S., Rahavian A., Garoosi M. (2023). Intravesical Gemcitabine versus Intravesical Bacillus Calmette-Guerin for the Treatment of Intermediate-Risk Non-Muscle Invasive Bladder Cancer: A Randomized Controlled Trial. Urol. J..

[39] Han M.A., Maisch P., Jung J.H., Hwang J.E., Narayan V., Cleves A., Hwang E.C., Dahm P. (2021). Intravesical gemcitabine for non-muscle invasive bladder cancer: An abridged Cochrane Review. Investig. Clin. Urol..

[40] Shelley M.D., Jones G., Cleves A., Wilt T.J., Mason M.D., Kynaston H.G. (2012). Intravesical gemcitabine therapy for non-muscle invasive bladder cancer (NMIBC): A systematic review. BJU Int..

[41] Addeo R., Caraglia M., Bellini S., Abbruzzese A., Vincenzi B., Montella L., Miragliuolo A., Guarrasi R., Lanna M., Cennamo G. (2010). Randomized phase III trial on gemcitabine versus mytomicin in recurrent superficial bladder cancer: Evaluation of efficacy and tolerance. J. Clin. Oncol..

[42] Hendricksen K., Witjes J.A. (2006). Intravesical gemcitabine: An update of clinical results. Curr. Opin. Urol..

[43] Kuperus J.M., Busman R.D., Kuipers S.K., Broekhuizen H.T., Noyes S.L., Brede C.M., Tobert C.M., Lane B.R. (2021). Comparison of Side Effects and Tolerability Between Intravesical Bacillus Calmette-Guerin, Reduced-Dose BCG and Gemcitabine for Non-Muscle Invasive Bladder Cancer. Urology.

[44] Study Details|A Study of TAR-200 in Combination with Cetrelimab, TAR-200 Alone, or Cetrelimab Alone in Participants with Non-Muscle Invasive Bladder Cancer (NMIBC) Unresponsive to Intravesical Bacillus Calmette-Guérin Who Are Ineligible for or Elected Not to Undergo Radical Cystectomy|ClinicalTrials.gov. https://clinicaltrials.gov/study/NCT04640623.

[45] Roumiguié M., Black P.C. (2022). Sequential Gemcitabine plus Docetaxel Is the Standard Second-line Intravesical Therapy for BCG-unresponsive Non-muscle-invasive bladder cancer: Pro. Eur. Urol. Focus.

[46] Tan W.S., McElree I.M., Davaro F., Steinberg R.L., Bree K., Navai N., Dinney C.P., O’Donnell M.A., Li R., Kamat A.M. (2023). Sequential Intravesical Gemcitabine and Docetaxel is an Alternative to Bacillus Calmette-Guérin for the Treatment of Intermediate-risk Non-muscle-invasive Bladder Cancer. Eur. Urol. Oncol..

[47] McElree I.M., Steinberg R.L., Martin A.C., Richards J., Mott S.L., Gellhaus P.T., Nepple K.G., O’Donnell M.A., Packiam V.T. (2022). Sequential Intravesical Gemcitabine and Docetaxel for bacillus Calmette-Guérin-Naïve High-Risk Nonmuscle-Invasive Bladder Cancer. J. Urol..

[48] McElree I.M., Steinberg R.L., Mott S.L., O’Donnell M.A., Packiam V.T. (2023). Comparison of Sequential Intravesical Gemcitabine and Docetaxel vs Bacillus Calmette-Guérin for the Treatment of Patients with High-Risk Non–Muscle-Invasive Bladder Cancer. JAMA Netw. Open.

[49] Yim K., Melnick K., Mott S.L., Carvalho F.L., Zafar A., Clinton T.N., Mossanen M., Steele G.S., Hirsch M., Rizzo N. (2023). Sequential intravesical gemcitabine/docetaxel provides a durable remission in recurrent high-risk NMIBC following BCG therapy. Urol. Oncol. Semin. Orig. Investig..

[50] A Randomized Phase III Trial of Intravesical BCG versus Intravesical Docetaxel and Gemcitabine Treatment in BCG Naive Non-Muscle Invasive Bladder Cancer (The BRIDGE Trial)—NCI. https://www.cancer.gov/research/participate/clinical-trials-search/v?id=NCI-2022-04864&r=1.

[51] Tomasz M. (1995). Mitomycin C: Small, fast and deadly (but very selective). Chem. Biol..

[52] Teus M.A., de Benito-Llopis L., Alió J.L. (2009). Mitomycin C in corneal refractive surgery. Surv. Ophthalmol..

[53] Bradner W.T. (2001). Mitomycin C: A clinical update. Cancer Treat. Rev..

[54] de Bruijn E.A., Sleeboom H.P., van Helsdingen P.J.R.O., van Oosterom A.T., Tjaden U.R., Maes R.A.A. (1992). Pharmacodynamics and pharmacokinetics of intravesical mitomycin C upon different dwelling times. Int. J. Cancer.

[55] Bolenz C., Cao Y., Arancibia M.F., Trojan L., Alken P., Michel M.S. (2006). Intravesical mitomycin C for superficial transitional cell carcinoma. Expert Rev. Anticancer Ther..

[56] Peyton C.C., Chipollini J., Azizi M., Kamat A.M., Gilbert S.M., Spiess P.E. (2019). Updates on the use of intravesical therapies for non-muscle invasive bladder cancer: How, when and what. World J. Urol..

[57] Järvinen R., Kaasinen E., Sankila A., Rintala E. (2009). Long-term efficacy of maintenance bacillus Calmette-Guérin versus maintenance mitomycin C instillation therapy in frequently recurrent TaT1 tumours without carcinoma in situ: A subgroup analysis of the prospective, randomised FinnBladder I study with a 20-year follow-up. Eur. Urol..

[58] Oddens J.R., Van Der Meijden A.P.M., Sylvester R. (2004). One immediate postoperative instillation of chemotherapy in low risk Ta, T1 bladder cancer patients. Is it always safe?. Eur. Urol..

[59] Sylvester R.J., Oosterlinck W., Van Der Meijden A.P.M. (2004). A single immediate postoperative instillation of chemotherapy decreases the risk of recurrence in patients with stage Ta T1 bladder cancer: A meta-analysis of published results of randomized clinical trials. J. Urol..

[60] Schmidt S., Kunath F., Coles B., Draeger D.L., Krabbe L.-M., Dersch R., Kilian S., Jensen K., Dahm P., Meerpohl J.J. (2020). Intravesical Bacillus Calmette-Guérin versus mitomycin C for Ta and T1 bladder cancer. Cochrane Database Syst. Rev..

[61] Bosschieter J., Nieuwenhuijzen J.A., van Ginkel T., Vis A.N., Witte B., Newling D., Beckers G.M., van Moorselaar R.J.A. (2018). Value of an Immediate Intravesical Instillation of Mitomycin C in Patients with Non-muscle-invasive Bladder Cancer: A Prospective Multicentre Randomised Study in 2243 patients. Eur Urol..

[62] Dalton J.T., Wientjes M.G., Badalament R.A., Drago J.R., Au J.L. Pharmacokinetics of Intravesical Mitomycin C in Superficial Bladder Cancer Patients. http://aacrjournals.org/cancerres/article-pdf/51/19/5144/2444855/cr0510195144.pdf.

[63] Cliff A.M., Romaniuk C.S., Parr N.J. (2000). Perivesical inflammation after early mitomycin C instillation. BJU Int..

[64] Elmamoun M.H., Christmas T.J., Woodhouse C.R.J. (2014). Destruction of the bladder by single dose Mitomycin C for low-stage transitional cell carcinoma (TCC)—Avoidance, recognition, management and consent. BJU Int..

[65] Tan W.S., Panchal A., Buckley L., Devall A.J., Loubière L.S., Pope A.M., Feneley M.R., Cresswell J., Issa R., Mostafid H. (2019). Radiofrequency-induced Thermo-chemotherapy Effect Versus a Second Course of Bacillus Calmette-Guérin or Institutional Standard in Patients with Recurrence of Non-muscle-invasive Bladder Cancer Following Induction or Maintenance Bacillus Calmette-Guérin Therapy (HYMN): A Phase III, Open-label, Randomised Controlled Trial. Eur. Urol..

[66] Stauffer P.R., van Rhoon G.C. (2016). Overview of bladder heating technology: Matching capabilities with clinical requirements. Int. J. Hyperth..

[67] Lammers R.J., Witjes J.A., Inman B.A., Leibovitch I., Laufer M., Nativ O., Colombo R. (2011). The role of a combined regimen with intravesical chemotherapy and hyperthermia in the management of non-muscle-invasive bladder cancer: A systematic review. Eur. Urol..

[68] Van Hattum J.W., Scutigliani E.M., Helderman R.F.C.P.A., Zweije R., Rodermond H.M., Oei A.L., Crezee J., Oddens J.R., De Reijke T.M., Krawczyk P.M. (2022). A scalable hyperthermic intravesical chemotherapy (HIVEC) setup for rat models of bladder cancer. Sci. Rep..

[69] Bahouth Z., Halachmi S., Moskovitz B., Nativ O. (2016). The role of hyperthermia as a treatment for non-muscle invasive bladder cancer. Expert Rev. Anticancer Ther..

[70] Plata A., Guerrero-Ramos F., Garcia C., González-Díaz A., Gonzalez-Valcárcel I., de la Morena J.M., Díaz-Goizueta F.J., del Álamo J.F., Gonzalo V., Montero J. (2021). Long-Term Experience with Hyperthermic Chemotherapy (HIVEC) Using Mitomycin-C in Patients with Non-Muscle Invasive Bladder Cancer in Spain. J. Clin. Med..

[71] Colombo R., DA Pozzo L.F., Lev A., Salonia A., Rigatti P., Leib Z., Servadio C., Caldarera E., Pavone-Macaluso M. (1998). Local Microwave Hyperthermia and Intravesical Chemotherapy as Bladder Sparing Treatment for Select Multifocal and Unresectable Superficial Bladder Tumors. J. Urol..

[72] Thomsen J.A., Nielsen Dominiak H., Lindgren M.S., Jensen J.B. (2021). Adverse events of hyperthermic intravesical chemotherapy for non-muscle invasive bladder cancer patients. Scand. J. Urol..

[73] Paroni R., Salonia A., Lev A., Da Pozzo L.F., Cighetti G., Montorsi F., Rigatti P., Colombo R., Paroni R., Salonia A. (2001). Effect of local hyperthermia of the bladder on mitomycin C pharmacokinetics during intravesical chemotherapy for the treatment of superficial transitional cell carcinoma. Br. J. Clin. Pharmacol..

[74] Colombo R., Da Pozzo L.F., Salonia A., Rigatti P., Leib Z., Baniel J., Caldarera E., Pavone-Macaluso M. (2003). Multicentric study comparing intravesical chemotherapy alone and with local microwave hyperthermia for prophylaxis of recurrence of superficial transitional cell carcinoma. J. Clin. Oncol..

[75] González-Padilla D.A., González-Díaz A., Guerrero-Ramos F., Rodríguez-Serrano A., García-Jarabo E., Corona-Lapuerta M., Rodríguez-Antolín A., Villacampa-Aubá F. (2021). Quality of life and adverse events in patients with nonmuscle invasive bladder cancer receiving adjuvant treatment with BCG, MMC, or chemohyperthermia. Urol. Oncol..

[76] Necchi A., Roumiguié M., Esen A.A., Lebret T., De Wit R., Shore N.D., Bajorin D.F., Krieger L.E.M., Kandori S., Uchio E.M. (2023). Pembrolizumab (pembro) monotherapy for patients (pts) with high-risk non–muscle-invasive bladder cancer (HR NMIBC) unresponsive to bacillus Calmette–Guérin (BCG): Results from cohort B of the phase 2 KEYNOTE-057 trial. J. Clin. Oncol..

[77] Chen W., Liu N., Yuan Y., Zhu M., Hu X., Hu W., Wang S., Wang C., Huang B., Xing D. (2022). ALT-803 in the treatment of non-muscle-invasive bladder cancer: Preclinical and clinical evidence and translational potential. Front. Immunol..

[78] Chang S., Chamie K., Hidalgo M., Kramolowsky E., Sexton W., Reddy S., Soon-Shiong P. (2022). PLLBA-01 FINAL CLINICAL RESULTS OF PIVOTAL TRIAL OF IL-15RΑFC SUPERAGONIST N-803 WITH BCG IN BCG-UNRESPONSIVE NON-MUSCLE INVASIVE BLADDER CANCER (NMIBC) CIS AND PAPILLARY COHORTS. J. Urol..

[79] Chamie K., Chang S.S., Gonzalgo M., Kramolowsky E.V., Sexton W.J., Bhar P., Reddy S.K., Soon-Shiong P. (2022). Final clinical results of pivotal trial of IL-15RαFc superagonist N-803 with BCG in BCG-unresponsive CIS and papillary nonmuscle-invasive bladder cancer (NMIBC). J. Clin. Oncol..

[80] Boorjian S.A., Alemozaffar M., Konety B.R., Shore N.D., Gomella L.G., Kamat A.M., Bivalacqua T.J., Montgomery J.S., Lerner S.P., Busby J.E. (2021). Intravesical nadofaragene firadenovec gene therapy for BCG-unresponsive non-muscle-invasive bladder cancer: A single-arm, open-label, repeat-dose clinical trial. Lancet Oncol..

[81] Lamm D., Brausi M., O’Donnell M.A., Witjes J.A. (2014). Interferon alfa in the treatment paradigm for non-muscle-invasive bladder cancer. Urol. Oncol..

[82] Grandi P., Darilek A., Moscu A., Pradhan A., Li R. (2023). Intravesical Infusion of Oncolytic Virus CG0070 in the Treatment of Bladder Cancer. Methods Mol. Biol..

[83] Ramesh N., Ge Y., Ennist D.L., Zhu M., Mina M., Ganesh S., Reddy P.S., Yu D.-C. (2006). CG0070, a conditionally replicating granulocyte macrophage colony-stimulating factor--armed oncolytic adenovirus for the treatment of bladder cancer. Clin. Cancer Res..

[84] Morizawa Y., Miyake M., Shimada K., Hori S., Tatsumi Y., Nakai Y., Tanaka N., Fujii T., Fujimoto K. (2018). Colony-stimulating factors detected in tumor cells and voided urine are potential prognostic markers for patients with muscle-invasive bladder cancer undergoing radical cystectomy. Res. Rep. Urol..

[85] Burke J.M., Lamm D.L., Meng M.V., Nemunaitis J.J., Stephenson J.J., Arseneau J.C., Aimi J., Lerner S., Yeung A.W., Kazarian T. (2012). A first in human phase 1 study of CG0070, a GM-CSF expressing oncolytic adenovirus, for the treatment of nonmuscle invasive bladder cancer. J. Urol..

[86] Deininger S., Törzsök P., Mitterberger M., Pallauf M., Oswald D., Deininger C., Lusuardi L. (2022). From Interferon to Checkpoint Inhibition Therapy-A Systematic Review of New Immune-Modulating Agents in Bacillus Calmette-Guérin (BCG) Refractory Non-Muscle-Invasive Bladder Cancer (NMIBC). Cancers.

[87] Packiam V.T., Lamm D.L., Barocas D.A., Trainer A., Fand B., Davis R.L., Clark W., Kroeger M., Dumbadze I., Chamie K. (2018). An open label, single-arm, phase II multicenter study of the safety and efficacy of CG0070 oncolytic vector regimen in patients with BCG-unresponsive non–muscle-invasive bladder cancer: Interim results. Urol. Oncol. Semin. Orig. Investig..

[88] Li R., Steinberg G.D., Uchio E.M., Lamm D.L., Shah P., Kamat A.M., Bivalacqua T., Packiam V.T., Chisamore M.J., McAdory J. (2022). CORE1: Phase 2, single-arm study of CG0070 combined with pembrolizumab in patients with nonmuscle-invasive bladder cancer (NMIBC) unresponsive to bacillus Calmette-Guerin (BCG). J. Clin. Oncol..

[89] Al Hussein Al Awamlh B., Chang S.S. (2023). Novel Therapies for High-Risk Non-Muscle Invasive Bladder Cancer. Curr. Oncol. Rep..

[90] Tan W.S., Grajales V., Contieri R., Hensley P., Bree K., Msaouel P., Guo C.C., Nogueras-Gonzalez G.M., Navai N., Dinney C.P. (2023). Bladder-sparing Treatment in Patients with Bacillus Calmette-Guerin-unresponsive Non-muscle-invasive Bladder Cancer: An Analysis of Long-term Survival Outcomes. Eur. Urol. Open Sci..

[91] Bandari J., Zummo J., Belani K., Brown E., Metcalf M., Nanayakkara N. (2022). Phase 1a/b safety study of intravesical instillation of TARA-002 in adults with high-grade non-muscle invasive bladder cancer (ADVANCED-1). J. Clin. Oncol..

